# IoT-Based Agro-Toolbox for Soil Analysis and Environmental Monitoring

**DOI:** 10.3390/mi14091698

**Published:** 2023-08-30

**Authors:** Eleftheria Maria Pechlivani, Athanasios Papadimitriou, Sotirios Pemas, Georgios Ntinas, Dimitrios Tzovaras

**Affiliations:** 1 Center for Research and Technology Hellas, Information Technologies Institute, 6th km Charilaou-Thermi Road, 57001 Thessaloniki, Greece; athpapa@iti.gr (A.P.); sopemas@iti.gr (S.P.); dimitrios.tzovaras@iti.gr (D.T.); 2 Hellenic Agricultural Organization-DIMITRA, Sustainable Agricultural Structures & Renewable Energy Resources Lab, Institute of Plant Breeding & Genetic Resources, 57001 Thessaloniki, Greece; gntinas@elgo.gr

**Keywords:** agriculture, IoT, soil analysis, environmental monitoring, 3D printed, tool, soil pH, soil moisture

## Abstract

The agricultural sector faces numerous challenges in ensuring optimal soil health and environmental conditions for sustainable crop production. Traditional soil analysis methods are often time-consuming and labor-intensive, and provide limited real-time data, making it challenging for farmers to make informed decisions. In recent years, Internet of Things (IoT) technology has emerged as a promising solution to address these challenges by enabling efficient and automated soil analysis and environmental monitoring. This paper presents a 3D-printed IoT-based Agro-toolbox, designed for comprehensive soil analysis and environmental monitoring in the agricultural domain. The toolbox integrates various sensors for both soil and environmental measurements. By deploying this tool across fields, farmers can continuously monitor key soil parameters, including pH levels, moisture content, and temperature. Additionally, environmental factors such as ambient temperature, humidity, intensity of visible light, and barometric pressure can be monitored to assess the overall health of agricultural ecosystems. To evaluate the effectiveness of the Agro-toolbox, a case study was conducted in an aquaponics floating system with rocket, and benchmarking was performed using commercial tools that integrate sensors for soil temperature, moisture, and pH levels, as well as for air temperature, humidity, and intensity of visible light. The results showed that the Agro-toolbox had an acceptable error percentage, and it can be useful for agricultural applications.

## 1. Introduction

In recent years, there have been significant advancements in the integration of smart technologies for precision farming within the agricultural domain [1]. These technologies can be seamlessly incorporated into conventional farming techniques, providing farmers with valuable insights to enhance their production [2]. Given that agriculture is the primary source of food and fundamental for national economies, it is imperative to monitor and preserve optimal cultivation conditions.

A notable innovation in this field has been the development of smart Internet of Things (ΙοΤ) mobile devices, which offer real-time monitoring and data collection capabilities [2]. The IoT plays a crucial role in enabling smart agriculture practices and contributes to increased production efficiency by effectively measuring soil, temperature, and humidity parameters in a cost-effective manner. The development of smart IoT mobile/portable devices has revolutionized precision agriculture, by addressing the need for real-time data acquisition [3]. Traditional farming methods often rely on manual sampling or visual inspection by farmers during frequent visits, which can be time-consuming and provide limited insights into field conditions [4]. In contrast, portable devices enable continuous data collection, offering a comprehensive understanding of crop health and facilitating interventions. These devices also contribute to more efficient and sustainable agriculture practices, supporting farmers in optimizing resource usage and improving crop yields during global population growth and changing climate patterns. Portable devices, equipped with advanced sensors, offer the capability to provide high-quality data through accurate measurements of environmental parameters. By utilizing information and communication technologies (ICT), such as the IoT, these devices can gather valuable data on farmers’ requirements and enable the implementation of precision farming practices in agriculture.

In an IoT agriculture inspector system, various parameters are important to collect and analyze to provide insights and recommendations for field management. These parameters can be separated to environmental parameters, which include air temperature, air humidity, intensity of visible light, and barometric pressure, and soil parameters, which include soil moisture, soil temperature, soil pH, electrical conductivity of soil, and soil nutrient level. As pH is a crucial parameter for various chemical and biological processes, obtaining accurate real-time pH data is therefore important for precise monitoring [5]. In addition, soil parameters are indicative of the health and fertility of agricultural land. Accurate measurement of these parameters plays a significant role in understanding the overall health and fertility of the soil. Soil pH, for instance, provides insights into its acidity or alkalinity, which in turn affects nutrient availability, metal solubility, and microbial activity [6,7]. Monitoring soil humidity allows farmers to assess moisture content, aiding in determining irrigation needs and avoiding issues such as overwatering and drought stress. Additionally, measuring soil temperature and air pressure provides valuable information about environmental conditions and their impact on crop growth and development [4,8,9]. Furthermore, air temperature and air humidity can affect crop growth and disease susceptibility [10]. Finally, monitoring the intensity of visible light can help growers optimize the amount of sunlight the crop receives; too much or too little sunlight can affect crop’s development and health [11].

Collecting and analyzing these measurements using an IoT agriculture inspector system can help growers make informed decisions about irrigation scheduling, nutrient management, pest control, and other critical field management practices. Therefore, an inspector system requires multiple sensors for data collection. Furthermore, an Agro-toolbox can support a RESTAPI architectural style for decision support systems, which can allow integrated nutrient management of open fields, as well as of hydroponics cultivations [12].

This paper introduces a novel IoT-based Agro-toolbox, a robust solution that synergizes with Industry 4.0. This do-it-yourself (DIY) toolbox leverages advanced commercial sensors, which are certificated and calibrated for use to provide comprehensive on-site measurement and monitoring for agricultural purposes. The proposed device finds applicability in field-based applications, involving uncomplicated communication steps with the user’s device. Its goal is to offer a simple method for integrating a customized and DIY comprehensive device with IoT technologies. Agro-toolbox stands as a systemic innovation, incorporating multiple sensors for soil analysis and environmental conditions. Housed inside a 3D printed box, these sensors collect data on key parameters, such as humidity, temperature, barometric pressure of air, intensity of visible light, soil moisture, and soil pH. The toolbox’s design, leveraging industrial design techniques, is ergonomic and customizable through simple 3D printing technologies. The proposed device provides data visualization capabilities via a Bluetooth-compatible Android device and an integrated IPS 2-inch display. The system is designed to enable farmers to optimize their agricultural practices, improve crop yields, and reduce waste and environmental impacts.

The paper is structured as follows: Section 2 provides a literature review with related work examples. Section 3 includes the materials and methods of the device, such as the hardware component specifications, usage firmware, additive manufacturing, prototyping, and calibration of pH sensor. Section 4 presents the experimental setup and testing of the Agro-toolbox. Section 5 includes the results and discussion. Finally, the study concludes in Section 6.

## 2. Related Work

This section examines various methodologies and combinations documented in the literature for soil element and pH measurements. Notably, these methodologies include different research domains, such as computer science and IoT, showcasing the interdisciplinary nature of this field.

Shirrmann et al. [13] presented the Veris Technologies’ Soil pH Manager™, an innovative sensor capable of real-time soil pH mapping. This sensor automates the collection of soil samples and measures pH directly while in motion across the field. Tests under controlled conditions revealed a strong linear relationship between the soil pH values obtained in standard laboratory settings and pH values recorded by the sensor. Ghazali et al. [14] proposed the Soil pH (SpHI) model, a method for estimating soil pH using satellite images and multiple regression equations. Tobiszewski et al. [15] reported that soil pH prediction can be achieved using an artificial neural network trained on a dataset of soil photos with known pH values. They also proposed an alternative method using a smartphone and classical indicators such as bromothymol blue, methyl red, and phenol red within the pH range of 1 to 13. Yin et al. [16] introduced emerging technologies known as plant wearables, which are attached to plants for in-situ monitoring of biological parameters, and wireless sensor networks for measuring various parameters including chemical signals in the soil, moisture, and pH. Vimal et al. [17] integrated a soil moisture sensor and pH sensor with complementary DHT11 sensor (for temperature and humidity) and LDR sensor (for light intensity) into an Arduino microcontroller. A GSM SIM800 device and Arduino Ethernet Shield were integrated to establish internet connectivity, thereby enabling real-time data transmission from a greenhouse environment to a mobile phone.

Hua et al. [18] presented a study of how artificial intelligent can help IoT applications, instead of the traditional cloud computing. This study defines edge computing (EC), highlights its appeal, and showcases AI’s role in optimizing EC and extending its application to various fields.

Pechlivani et al. [12] proposed a robust decision support system (DSS) with user-centric, cloud-based farm management system. It utilized real-time data from digital and space-based technologies, employing artificial intelligence (AI) algorithms and a user-friendly interface for data collection and remote sensing. It facilitates proactive measures and automated decision-making for integrated pest management (IPM) and integrated nutrient management (INM), offering a promising solution for the challenges of Agriculture 4.0, while fostering sustainable farming practices.

Although numerous systems have been proposed for obtaining soil measurements, none of them feature an ergonomic 3D-printed housing like the one proposed in this study. The Agro-toolbox, as proposed, enables in situ monitoring of soil conditions, with real-time measurements accessible via both the embedded display on the toolbox and the accompanying mobile application. By doing so, the Agro-toolbox aims to offer a variety of features and measurements, surpassing existing systems in terms of user-friendliness, convenience, and efficiency.

## 3. Methodology

**Figure 1:**
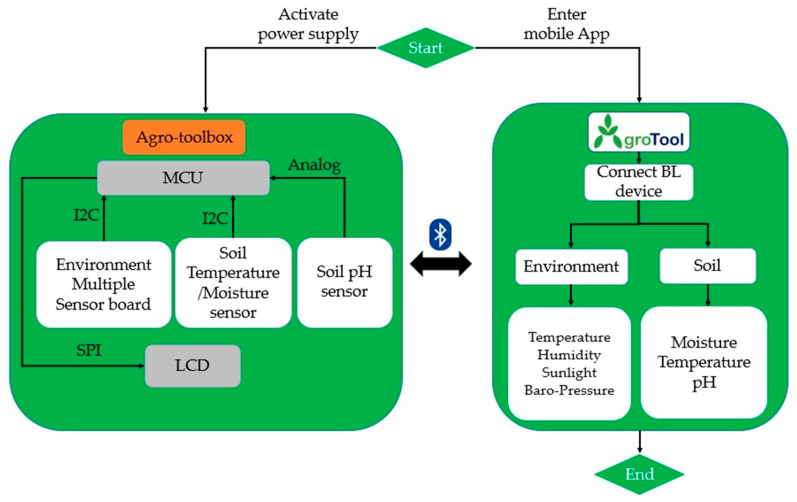
Architectural diagram of the Agro-toolbox.

This section outlines the hardware design of the proposed device, such as the sensors and microcontroller. Additionally, it presents a firmware flowchart and mobile application implemented for the Agro-toolbox. Furthermore, the additive manufacturing method and the prototyping-assembly of the device are shown. Finally, the experimental setup and the benchmarking of the proposed device are presented. Figure 1 shows the architectural diagram and the overall operation of the proposed IoT based Agro-toolbox. The MCU of the device collects all the sensor data via the I2C protocol and analog signals. These data can be viewed by the user via an LCD module or the display of an Android smartphone via the developed mobile app. In the mobile app, the measurements from the environmental and soil sensors are presented in two different screen options.

### 3.1. Hardware Design

To reduce the need for individual sensors, integrated sensor boards capable of performing multiple measurements were utilized. These included the Multiple Function Sensor Development Tools Sensor Boards from Omron Electronics and the Adafruit STEMMA board. The Omron board consists of an air temperature/humidity sensor, with a measurements range of −40 to 125 °C for temperature and 0 to 100% for humidity, a MEMS digital barometric pressure sensor with atmospheric pressure range of 30 kPa to 110 kPa, and an ambient light sensor with a measurement range of 0.01 lux to 83,000 lux. The Adafruit STEMMA board features a soil temperature and a soil moisture sensor, which are combined to give readings in the range of 200 (very dry) to 2000 (very wet) or each feature individually. The DFRobot Analog Spear Tip pH meter is the only standalone sensor, with a gauge range of 0–10 in pH scale. Table 1 presents the specifications for all sensors.

The data collected by these sensors are sent to an Adafruit ESP32 V2 (MANSFIELD, TX US) feather microcontroller, in order to configure the system’s firmware development. The Bluetooth range of the proposed device, utilizing ESP32 V2 feather microcontroller capabilities, reaches a maximum of 10 m. This is achieved with a 2.4 GHz wireless protocol, and the device has a storage capacity of 520 kB for data and instructions [19,20]. Sensor measurements are displayed on an IPS SPI LCD module with a 2-inch diagonal and a resolution of 240 × 320. The chosen power source for the proposed device is a 20,000 mAh power bank, capable of supporting several hours of agricultural inspection. The selected model is a Veger power bank with Quick Charge 3.0 compatibility, which can be easily replaced if it ceases to function. The specifications of these components are detailed in Table 2.

In order for Agro-toolbox to be considered a low-cost device and be compared with alternatives, Table 3 presents the costs of the commercial hardware parts according to the official manufacturer sites. The total cost of the Agro-toolbox’ hardware system was calculated at EUR 299.51.

### 3.2. Firmware and Mobile Application Development

#### 3.2.1. Firmware

**Figure 2:**
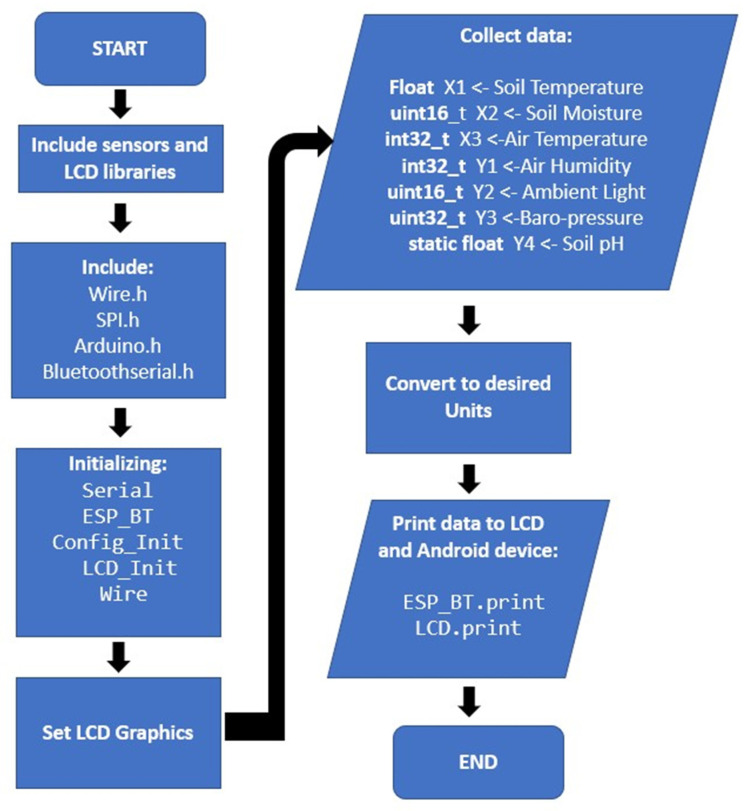
Agro-toolbox firmware flowchart.

The firmware process of the Agro-toolbox utilizes Arduino IDE software. The developed code consists of parts for collecting sensor data via I2C or SPI communication protocols, printing the data to the LCD display, sending them to the mobile app, and finally coding parts for the configuration of the display’s interface. Multiple libraries for the sensors and the LCD display were added to IDE, in order to complete the firmware development of the device, such as SPI and wire libraries for the communication of the sensors with the microcontroller. Additionally, the Bluetooth library was used to connect the Agro-toolbox with a smartphone device. A flowchart of the tool’s firmware development is shown in Figure 2. The sensors send the collected data via SPI or I2C to the ESP32 and then the microcontroller sends them to the mobile app and the LCD module. Simultaneously, the ESP32 creates the interface for the LCD display.

#### 3.2.2. Mobile Application

**Figure 3:**
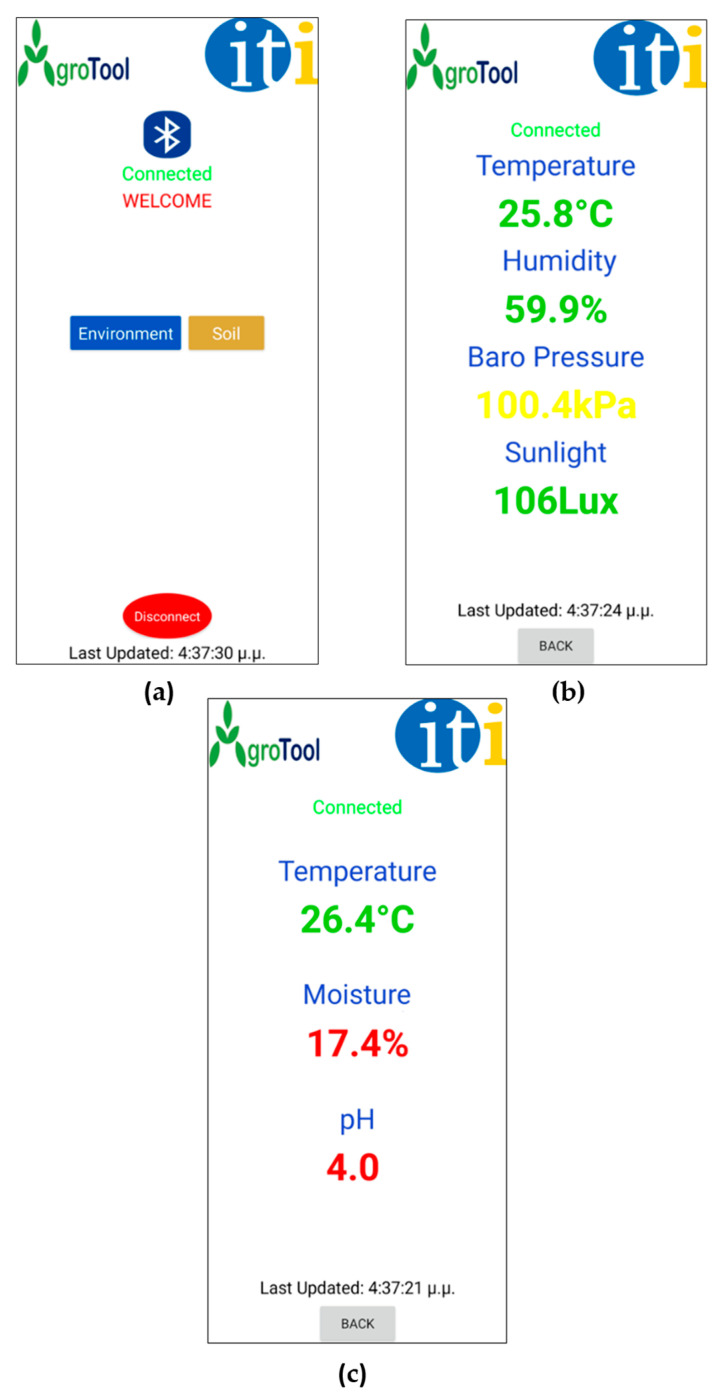
(**a**) Home screen of mobile app; (**b**) Environmental sensor screen; (**c**) Soil sensor screen.

The mobile application was developed using MIT App Inventor, an open-source web-based platform. The method that was followed for the App’s development was to design the main buttons, texts, and logos using the “Designer” section via Horizontal Arrangement layout in the MIT App inventor. Furthermore, in the “Blocks” section of MIT App inventor, all the required blocks were added, such as the variables, logic, math, lists, texts, control, and procedures blocks. The main development of the mobile application was achieved in the “Blocks” section, as from there the designer can add all the required instructions about the developed mobile application. The mobile application was designed to integrate all of the sensor measurements into a user-friendly interface. The application’s name is “AgroTool” and a specific logo was designed in order to increase its prototyping. Figure 3 shows the application’s interface in an android device.

In the home screen (Figure 3a), the user connects the device to an android device via Bluetooth. To access the list of the available Bluetooth devices the user touches the Bluetooth symbol. If the Bluetooth of the android device is not enabled, a notification is shown on the screen. Once the user is connected, the status switches from disconnected to connected. Then the user can live monitor the field’s soil parameters from the soil sensor screen (Figure 3b) by touching the button “Soil” and the field’s environmental parameters from the environmental sensor screen (Figure 3c) by touching the button “Environment”. By pressing the button “Back”, the user can return to the home screen, and the button “Disconnect” will disconnect the Agro-toolbox from the android device. There is a time stamp at the bottom of every screen that shows the elapsed time since starting the connection with the Agro-toolbox.

### 3.3. Additive Manufacturing

The Agro-toolbox is based on a holistic construction of an assembled device based on the digital manufacturing process 3D printing, according to which customized parts can be printed rapidly and in complex geometries. These parts are the main components that make up the shell of the device. They include parts for the housing of electronics and components that enable user interaction. The 3D design was accomplished using Solidworks software, a computer-aided design (CAD) program. The overall design consists of three main parts that form the final system. The Agro-toolbox is assembled with multiple components, to facilitate easy adjustment and replacement of electronic components such as the sensors and power bank.

The first main part is the “Smart handle,” which houses the PCB, power bank, display screen and environmental sensors, including the air humidity and temperature sensors, ambient light sensor, and finally the barometric pressure sensor. The “Smart handle” consists of two 3D printed parts: one is perfectly designed to match the precise dimensions of the commercialized electronic components, ensuring a perfect fit and stability during use, while the other part functions as a cap. These two parts are joined together using six screws, three on each side. Additionally, two small 3D printed parts are attached with screws to securely hold the power bank in place.

The second main part is the “Adjustable pole” which allows ergonomic usage of the system and cable protection. This pole consists of two 50 cm poles, one fitting inside the other. It features holes along its length, providing the user with the flexibility to adjust the height of the system according to their own stature, ensuring comfortable use. Furthermore, the “Adjustable pole” acts as a protective tube for the cables that connect the ground sensors to the PCB.

The third main part is the “Ground sensor mounting kit” which houses the two ground sensors. This part comprises three subparts. The first subpart is designed to accommodate the sensors securely, the second subpart acts as a cap and socket for the “Adjustable pole” and the third subpart serves as a protective cap for the sensors. To enhance stability and withstand the pressure when the sensors are placed in the ground to obtain measurements, one sensor is screwed into a built-in thread on the subpart, while the other is secured with two screws.

The connection between these three main parts and their respective sub-parts is established using screws of different sizes. The design of the device takes into consideration its utilization by users in a standing position, while also allowing for length adjustment using a single screw. Additionally, the device is lightweight, enabling effortless transportation to the desired measurement location in the field. It operates on a battery, providing the user with easy access for charging or replacement. Lastly, the design ensures that the user can easily access the electronic components for replacement in case of damage.

The fused filament fabrication (FFF) 3D printing technique was employed for the additive manufacturing process of all the 3D printed parts. This technique was chosen for its rapid and cost-effective nature [21], making it ideal for the development of a DIY Agro-toolbox. Since the Agro-toolbox will be used in an external environment exposed to sunlight and high temperatures, especially during the summer period, PETG (Poly Ethylene Terephthalate Glycol) was selected as the construction material. PETG is known for its durability and ability to withstand high temperatures [22,23]. The PETG material used was in filament form with a diameter of 1.75 mm and yellow color. It was utilized as the feedstock material for the RAISE3D Pro3 Plus FFF 3D printer. The slicing software used for setting the 3D printing parameters was IdeaMaker. Further details regarding the specific 3D printing parameters for each part can be found in the accompanying table. The 3D printing parameters of the Agro-toolbox parts are shown on Table 4.

Table 5 presents information related to the material quantity used for creating each part, alongside cost details. The table presents data on the duration of printing for each part and the material weight required for their manufacturing. The cost estimation for PETG filament is based on a 1 kg price of EUR 18.54 (excluding VAT). The power consumption of the 3D Printer was arbitrarily set at 120 Watts, serving as an approximation for total part cost computation. Moreover, the assumed cost for one kilo-watt-hour (1 kWh) was the EUR 0.39 current in Greece.

The Cost of material and Cost of energy were calculated using the following equations:

(1) $$Cost\;of\;material=\frac{\mathrm{Filament}\;\mathrm{price}\;\left(1000\;g\right)\times \;\mathrm{Used}\;\mathrm{filament}\;\left(\mathrm{for}\;\mathrm{each}\;\mathrm{part}\right)}{1000}$$

(2) $$Cost\;of\;energy=E\times Price\;of\;1\;\mathrm{kWh}\;\left(0.39€\right)$$

(3) $$E=\frac{P\left(W\right)\times t\left[\left(\frac{\min}{60}\right)+h\right]}{1000\frac{W}{\mathrm{kW}}}$$

(4) Unit cost=Cost of material+Cost of energy

The provided data indicate that the total cost for 3D Printing an IoT-based Agro-toolbox was EUR 21.24 excluding VAT, with the total weight of the 3D printed components being 908.3 g. It is worth noting that this cost is approximate and subject to variation based on factors such as electricity rates, filament costs, printing parameters, 3D printer power consumption, etc.

### 3.4. Assembly and Prototyping

**Figure 4:**
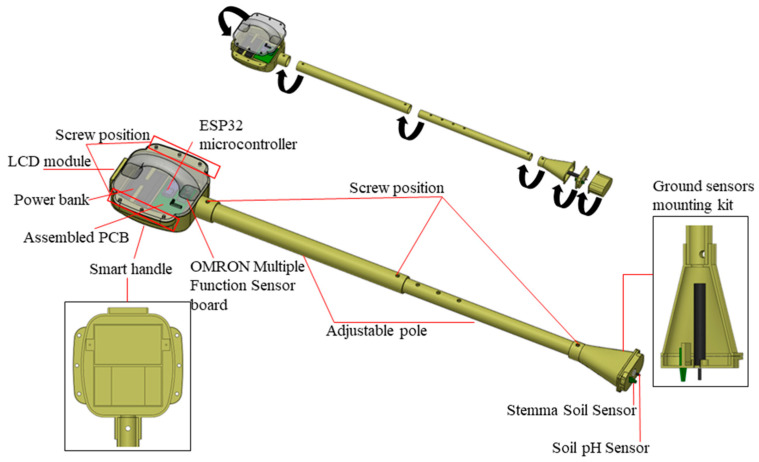
Agro-toolbox final assembly 3D model.

After completing the 3D printing process of the Agro-toolbox parts and subparts as described above, the assembly phase involved integrating the electronic components into their designated positions. Figure 4 illustrates the final assembly in a 3D model, showcasing the placement of the electronic components secured by metal screws.

**Figure 5:**
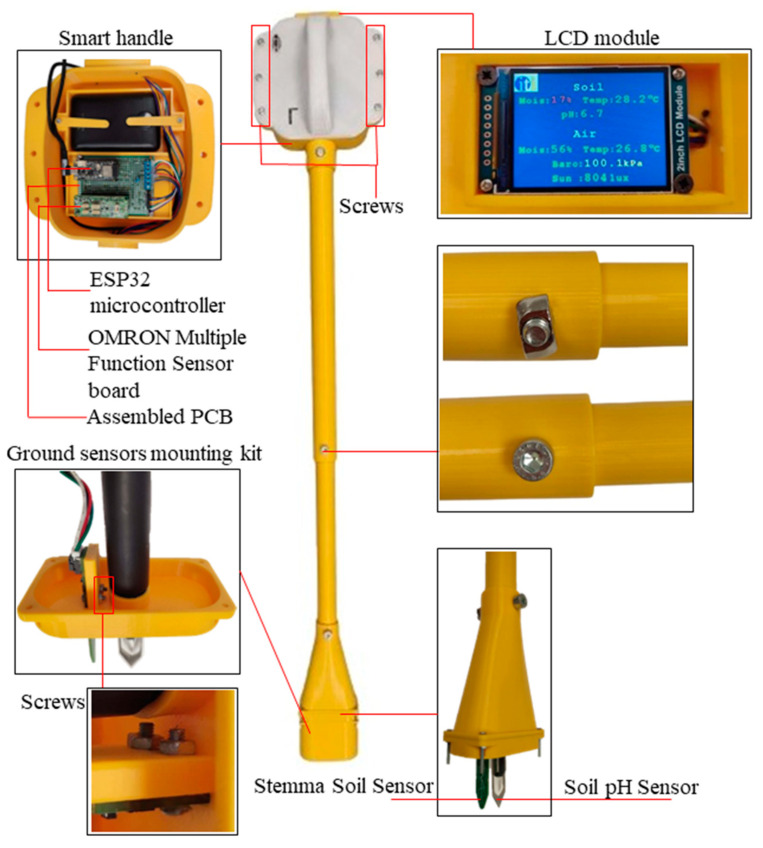
Prototyping of Agro-toolbox.

The screws were employed to connect the three main parts, as well as to secure the smart handle’s cap, the Stemma soil sensor, the assembled PCB, and the LCD module. Additionally, screws were utilized to hold the power bank securely in place, ensuring stability during usage. The ground sensor mounting kit was also connected using screws, along with its protective cap. Furthermore, during the prototyping and assembly process, the appropriate cable lengths for connecting the ground sensors to the assembled PCB were determined. Figure 5 presents the final prototyping of the proposed device.

### 3.5. Calibration Method for the pH Sensor

Calibration can be achieved using the 4.00 pH standard solution which is included in the sensor’s package and one glass of bottled water. The calibration steps are described below [24].

The pH probe is inserted into the 7.00 standard buffer solution for one minute. The pH value is displayed by accessing the serial monitor in the Arduino IDE. It should be ensured that the error is below 0.3. The displayed value is taken note of and compared to 7.00. The disparity should be entered as the “Offset” variable in the provided code sample. It is advisable for the pH probe to be cleaned using pure water or distilled water.

Afterwards, any remaining water residue is absorbed using filter paper. The pH probe is immersed into the 4.00 pH standard solution, waiting for a minimum of one minute. The serial monitor in the Arduino IDE is opened, and the potentiometer on the transmitter board is adjusted to fine-tune the gain until the displayed value stabilizes around 4.00.

Cleaning the pH probe with pure water or distilled water is recommended. Then, the residual water is absorbed with filter paper. Once these steps have been completed, the calibration process is finished. The soil pH sensor is now ready to be utilized for actual measurements.

## 4. Experimental Setup and Testing

The testing of the proposed device was performed inside a pilot greenhouse of Sasrer Lab, ELGO-DIMITRA in Thermi Thessaloniki, Greece (40°32′17.4″ N, 22°59′58.2″ E). The experimental greenhouse included two spaces, one above ground for plant production and one below ground for fish production, following a vertical production approach to achieve better land and energy use efficiency. The water temperature in the cultivation tanks, greenhouse air temperature and RH, as well the intensity of visible light were monitored with a Delta-T datalogger. The system consists of a rocket crop (*Eruca Sativa*) placed in a large floating tank, which contains soil with substrate of peat or perlite [25]. Four sets of case studies were conducted. Case 1 and case 2 included rocket with substrate of peat and pearlite correspondingly, and the floating system was infused with a Hoagland solution that provides every essential nutrient for plant growth [26], with 2.2 μS/cm^2^ electrical conductivity. Case 3 and case 4 had substrates of peat and pearlite, correspondingly, with a solution derived from fish waste with 0.5 μS/cm^2^ electrical conductivity and lower nutrient content. The proposed device was benchmarked against the Meter Teros 12 tool, known for its capabilities for measuring soil temperature and moisture. As for pH measurement, a Hanna HI98129 was utilized. Finally, the environmental parameters of the Agro-toolbox were benchmarked against the Delta-T datalogger weather station, except the barometric pressure sensor that had a standardized value range for applications in low altitude.

**Figure 6:**
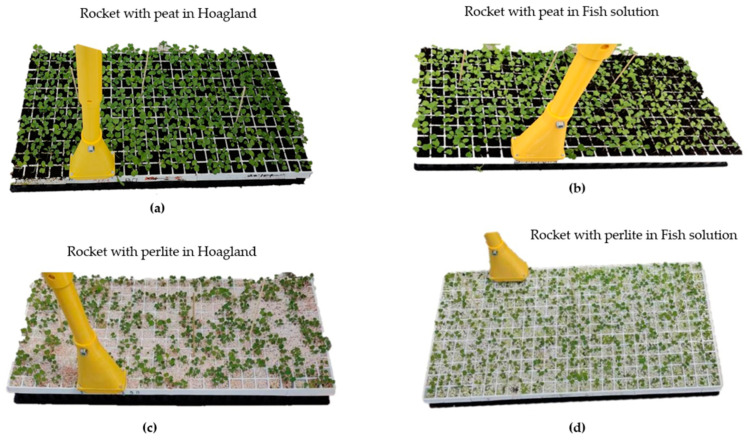
Experimental setup with Agro-toolbox for (**a**) Case1: Rocket with peat in Hoagland solution; (**b**) Case 3: Rocket with peat in Fish solution; (**c**) Case 2: Rocket with perlite in Hoagland solution; (**d**) Case 4: Rocket with perlite in Fish solution (Sasrer Lab, Thermi, ELGO-DIMITRA).

**Figure 7:**
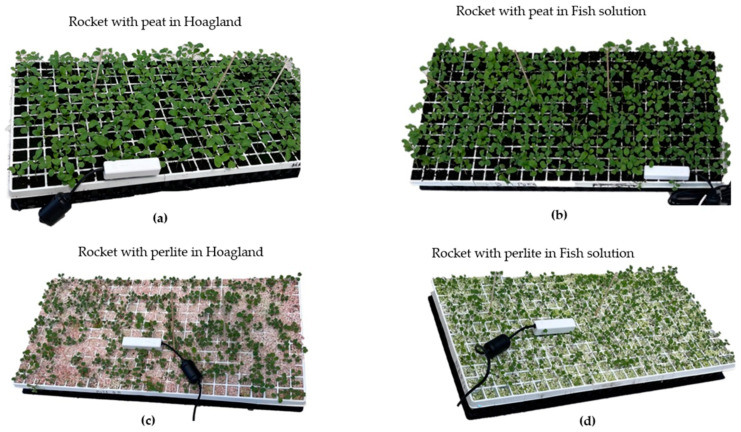
Experimental setup with Meter Teros 12 for (**a**) Case1: Rocket with peat in Hoagland solution; (**b**) Case 3: Rocket with peat in Fish solution; (**c**) Case 2: Rocket with perlite in Hoagland solution; (**d**) Case 4: Rocket with perlite in Fish solution (Sasrer Lab, Thermi, ELGO-DIMITRA).

Each measurement involved one specific spot in every case study, with each test lasting five minutes after removing the pot from the floating system. The measurement processes are presented in Figure 6 (Agro-toolbox) and Figure 7 (Meter Teros 12). The collected data were processed to compute average values for each case, encompassing parameters such as soil temperature, soil moisture, and soil pH.

## 5. Results and Discussion

The measurements for soil parameters took place in every cultivation tray sample, with a duration of 5 min and with the tray taken out of the floating system. From the collected data, the average values were calculated for each case. The measurements included soil temperature, soil moisture, and soil pH. Three sample measurements were taken for each tray.

As shown in Table 6 and Table 7, the exported data for the compared tools presented an error of 0.69% for the average soil temperature value, 4.24% for the average soil moisture value, and 0.71% for the average soil pH value. The percentage error remained low for case 1, expect soil moisture, which was slightly different in relation to temperature and pH but still within an acceptable range (<10%).

Sample values from the Agro-toolbox and the commercial tools case 2 are presented in Table 8 and Table 9, respectively. From Table 6 and Table 7, the percentage error for temperature was 2.72%, for moisture 16.80%, and for pH 0.3%. In this case, the measurements for temperature and moisture were lower than the first sampling measurement, because of the perlite’s absorption properties. The soil pH was higher in this tray sample and the error was reduced by half. The percentage error for moisture increased a lot, which may have been due to the roughness of the perlite, which made it difficult for the soil moisture sensor to accurately measure this sample.

The measurements of the Agro-toolbox and of the commercial tools for case 3 are presented in Table 10 and Table 11, respectively. The percentage error in this case was 7.02% for temperature, 20.45% for moisture, and 0.71% for soil pH. In these measurements, the error for temperature and pH was approximately the same as the sample for rocket with peat in Hoagland solution, and the moisture error increased a lot because of the peat’s absorption capacity.

The exported data from the Agro-toolbox and the commercial tools for case 4 are presented in Table 12 and Table 13, respectively. The percentage error for temperature remained at the same level with value of 9.34%, for moisture decreased by 0.83%, and the soil pH’s error reduced by 0.6%. In this case, moisture presented a large difference, related to the error value for the sample tray of rocket with perlite in Hoagland solution. The soil temperature error slightly increased and the soil pH presented variations of 0.3–0.71%.

The environmental parameter testing was performed inside a pilot greenhouse of Sasrer Lab, ELGO-DIMITRA, except intensity of visible light parameter, which required an outdoor measurement and the benchmarking values exported from Delta-T datalogger for the specific day and time of the test. Table 14 and Table 15 present all the benchmarked environmental measurements as they were exported from the Agro-toolbox and Delta-T datalogger, respectively. The Agro-toolbox took 3 measurements every 10 min, from 11:10 AM to 11:30 AM for every environmental parameter and the average value was calculated.

The calculated percentage error for air temperature was 0.85%. The measurements naturally increased as time passed for both devices. The air humidity percentage error was calculated as 4.12%. In this case, the humidity reduced with the increase in air temperature. Finally, for the intensity of visible light, the calculated error percentage was 9.49% and the measurements naturally increased as noon approached.

The proposed Agro-toolbox demonstrated an acceptable error percentage in all soil testing cases, which was consistently <10%, except for the moisture measurements in cases 2 and 3. The data values for soil temperature indicated that Agro-toolbox could measure accurately under different conditions, with a good result for error. For soil moisture measurements, Agro-toolbox showed more effective results at lower values (<30%), while the higher error percentage in cases 2 and 3 may be attributed to the sensor’s precision, which is ±2%, as well as the small depth of the measured pot. Case 1 and case 4 presented the best conditions for Agro-toolbox to measure accurately related to the commercial tools. Lastly, the soil pH sensor exhibited the most efficient soil measurements, with an error percentage of under 0.75% in all cases.

As for the environmental parameters, the Agro-toolbox presented even better error values especially for air temperature and humidity. The high error value for the intensity of visible light was due to the small testing surface of the ambient light sensor compared to the light sensor of the Delta-T datalogger.

## 6. Conclusions

In conclusion, the IoT-based Agro-toolbox developed for measuring soil and environmental parameters has proven to be a viable and efficient alternative to commercial tools. Through careful calibration and benchmarking against recognized commercial tools, the proposed tool demonstrated an acceptable level of accuracy in its final results. This DIY, portable, and user-friendly device is accessible to individuals lacking technological expertise, including farmers, citizen farmers, and farm to fork stakeholders. The total cost remains below EUR 400. It is easy to fabricate with 3D printed techniques and the selected material (PETG) has proven its waterproof and UV resistance abilities. Moreover, the selected 3D printed material (PETG) proved its waterproof ability after some hours of onsite testing, as the Agro-toolbox remained unaffected by the floating system’s moisture. The proposed device could effectively measure soil pH, with an error <0.75%. Finally, the air temperature and humidity presented very efficient values. The mobile application worked properly in the experimental area and the proposed device remained intact throughout the duration of the measurements. Thus, the Agro-toolbox device can reliably measure in real case scenario applications. The shortcomings of Agro-toolbox are mainly associated with the soil moisture and ambient light sensors, which presented high calculation errors in some cases. Further study and experiments will be conducted focusing on these sensors, to ensure effective measurements across all case studies.

The development of this DIY tool offers numerous advantages, particularly in the realm of its ease construction and accessibility. By utilizing readily available components and simple 3D printing construction methods, the tool can be assembled at a fraction of the cost of its commercial counterparts. Additionally, the Agro-toolbox empowers individuals to have direct control over their soil monitoring processes. By being able to measure crucial parameters such as soil pH, temperature, and moisture, as well as air temperature, humidity, and intensity of visible light on their own, users can gain valuable insights into the health of their soil and environment and make informed decisions about watering, fertilization, and plant selection. This increased understanding could ultimately contribute to more efficient and sustainable agricultural practices. While the Agro-toolbox may not achieve the same level of precision as high-end commercial instruments, the acceptable level of error demonstrated in the benchmarking process makes it a reliable and practical solution for many users. It can provide accurate and consistent measurements within an acceptable range, allowing users to make informed decisions regarding their soil and environment management practices. Finally, it can be utilized for application scenarios such as within a vineyard or an olive orchard.

Future improvements to the proposed tool could involve exploring alternative sensor options and functions, such as electrical conductivity measurements to further enhance accuracy and applicability. Implementation in greenhouse hydroponic cultivation substrates would provide precise information on soil and substrates salinity levels and also the availability of specific nutrients for crops of high commercial interest. Additionally, incorporating wireless connectivity and data logging capabilities would allow for convenient data collection and analysis over time, enabling users to monitor and track changes in soil and environment conditions more effectively.

## Figures and Tables

**Table 1:** System sensor’s specifications.

Sensor Name	Part Number	Parameter	Value	Units
Air Temperature Sensor		Resolution	0.01	°C
		Specified Range	−40 to 125	°C
	SHT30-DIS-B Sensirion	Response time	>2	s
		Accuracy tolerance	±0.2	°C
		Long-Term Drift	<0.03	°C/y
Air Humidity Sensor		Resolution	0.01	%RH
	SHT30-DIS-B Sensirion	Specified Range	0 to 100	%RH
		Response time	8	s
		Accuracy tolerance	±2	%RH
		Long-Term Drift	<0.25	%RH/y
Ambient Light Sensor		Illuminance max value	83,865.6	lux
	OPT3001DNP Texas Instruments	Resolution	0.01	lux
		Operating voltage	1.6–3.6	V
MEMS digital barometric pressure sensor		Resolution	0.06	Pa
	2SMPB-02E OMRON	Specified Range	30 to 110	kPa
		Accuracy tolerance	±50 (0–65 °C) ±80 (−20–0 °C)	Pa
Adafruit STEMMA Soil Sensor		Temperature precision	±2	°C
	Adafruit 4026	Moisture range	200–2000	
		Operating voltage	3.0–5.0	V
Soil pH sensor		Measuring Range	0–10	pH
		Accuracy	±0.1	pH
	SEN0249 DFRobot	Operating Temperature	5–60	°C
		Operating Voltage	5.00	V

**Table 2:** Hardware part specifications.

Name	Part Number	Parameter	Value	Units
ESP32 V2 feather Wi-Fi, Bluetooth, I2C, SPI		Flash RAM + PS RAM	8 + 2	MB
	Adafruit 5400	SRAM	520	kB
		CPU clock frequency	240	MHz
Waveshare LCD IPS Display module	17,344	Resolution	240 × 320	pixel
		Aspect Ratio	16:9	units
		Diagonal	2	inch
Veger Power bank	VP3008PD	Capacity	20.000	mAh
		Charging Power	22.5	W

**Table 3:** Cost of hardware parts.

Name	Part Number	Quantity	Cost (without VAT)
ESP32 V2 feather	Adafruit 5400	1	EUR 17.96
Waveshare LCD IPS Display module	17,344	1	EUR 11.00
Veger Power bank	VP3008PD	1	EUR 73.40
Adafruit STEMMA Soil Sensor	Adafruit 4026	1	EUR 6.75
Soil pH sensor	SEN0249 DFRobot	1	EUR 93.06
Multiple Function Sensor Development Tools Sensor Eval Board Arduino	2JCIE-EV01-AR1	1	EUR 97.34
Total Cost			EUR 299.51

**Table 4:** The 3D printing parameters of the Agro-toolbox parts.

3D Printed Part	3D Printed Subparts	AM Technique	Material	Layer Height	Infill	Nozzle/Build Platform Temperature
1. Smart handle	Smart handle base	FFF	PETG	0.2 mm	20%	240/80 °C
	Smart handle cap	FFF	PETG	0.2 mm	20%	240/80 °C
	Power bank holders	FFF	PETG	0.2 mm	20%	240/80 °C
2. Adjustable pole	Pole 1	FFF	PETG	0.2 mm	20%	240/80 °C
	Pole 2	FFF	PETG	0.2 mm	20%	240/80 °C
3. Ground sensors mounting kit	Ground sensors base	FFF	PETG	0.2 mm	20%	240/80 °C
	Ground sensors cap	FFF	PETG	0.2 mm	20%	240/80 °C
	Ground sensors protective cap	FFF	PETG	0.2 mm	20%	240/80 °C

**Table 5:** The 3D printed parts bill of materials.

3D Printed Part	3D Printed Subparts	Used Filament (g)	Printing Time	Cost of Energy (€)	Cost of Material (€)	Unit Cost (€)
1. Smart handle	Smart handle base	336.2	38 h 25 min	1.797	6.23	8.03
	Smart handle cap	171.4	18 h 54 min	0.884	3.17	4.06
	Power bank holders	3.1	22 min	0.011	0.05	0.06
2. Adjustable pole	Pole 1	106	14 h 13 min	0.443	1.96	2.40
	Pole 2	136.8	17 h 57 min	0.560	2.53	3.09
3. Ground sensors mounting kit	Ground sensors base	20.4	2 h 48 min	0.087	0.37	0.46
	Ground sensors cap	80.9	10 h 9 min	0.316	1.49	1.81
	Ground sensors protective cap	53.5	6 h 23 min	0.298	0.99	1.29

**Table 6:** Collected data from Agro-toolbox for case 1.

Measurement	Temperature (°C)	Moisture (%)	pH
1	33.00	30.70	5.60
2	32.90	29.50	5.60
3	32.60	28.20	5.70
Avg	32.83	29.46	5.58

**Table 7:** Collected data from Teros 12 and Hanna HI98129 for case 1.

Measurement	Temperature (°C)	Moisture (%)	pH
1	32.90	28.30	5.70
2	33.10	28.00	5.60
3	33.20	28.50	5.70
Avg	33.06	28.26	5.62

**Table 8:** Collected data from Agro-toolbox for case 2.

Measurement	Temperature (°C)	Moisture (%)	pH
1	32.50	29.40	6.60
2	32.30	29.90	6.60
3	31.70	29.50	6.50
Avg	32.16	29.60	6.54

**Table 9:** Collected data from Teros 12 and Hanna HI98129 for case 2.

Measurement	Temperature (°C)	Moisture (%)	pH
1	32.90	25.30	6.60
2	33.10	25.20	6.60
3	33.20	25.50	6.50
Avg	33.06	25.33	6.56

**Table 10:** Collected data from Agro-toolbox for case 3.

Measurement	Temperature (°C)	Moisture (%)	pH
1	30.20	41.40	5.60
2	30.10	40.70	5.70
3	29.90	39.80	5.80
Avg	30.06	40.63	5.66

**Table 11:** Collected data from Teros 12 and Hanna HI98129 for case 3.

Measurement	Temperature (°C)	Moisture (%)	pH
1	32.30	33.90	5.60
2	32.50	33.70	5.70
3	32.20	33.60	5.70
Avg	32.33	33.73	5.62

**Table 12:** Collected data from Agro-toolbox for case 4.

Measurement	Temperature (°C)	Moisture (%)	pH
1	29.60	29.90	6.70
2	29.90	30.40	6.60
3	30.10	23.50	6.70
Avg	29.88	27.93	6.62

**Table 13:** Collected data from Teros 12 and Hanna HI98129 for case 4.

Measurement	Temperature (°C)	Moisture (%)	pH
1	33.00	25.10	6.70
2	32.90	29.90	6.60
3	33.00	28.10	6.50
Avg	32.96	27.70	6.58

**Table 14:** Collected data from Agro-toolbox for environmental parameters.

Measurement	Temperature (°C)	Humidity (%)	Visible Light Intensity (Lux)
1	30.10	71.30	30,560.00
2	30.50	69.70	32,223.00
3	31.00	65.50	34,330.00
Avg	30.53	68.83	32,371.00

**Table 15:** Collected data from Delta-T datalogger for environmental parameters.

Measurement	Temperature (°C)	Humidity (%)	Visible Light Intensity (Lux)
1	29.90	74.30	33,670.90
2	30.30	73.00	35,443.00
3	30.60	67.70	37,215.20
Avg	30.27	71.67	35,443.03

## Data Availability

Data available on request.
